# Human monoclonal antibodies block the binding of SARS-CoV-2 spike protein to angiotensin converting enzyme 2 receptor

**DOI:** 10.1038/s41423-020-0426-7

**Published:** 2020-04-20

**Authors:** Xiangyu Chen, Ren Li, Zhiwei Pan, Chunfang Qian, Yang Yang, Renrong You, Jing Zhao, Pinghuang Liu, Leiqiong Gao, Zhirong Li, Qizhao Huang, Lifan Xu, Jianfang Tang, Qin Tian, Wei Yao, Li Hu, Xiaofeng Yan, Xinyuan Zhou, Yuzhang Wu, Kai Deng, Zheng Zhang, Zhaohui Qian, Yaokai Chen, Lilin Ye

**Affiliations:** 1 Institute of Immunology, PLA, Third Military Medical University, Chongqing, 400038 China; 2 State Key Laboratory of Veterinary Biotechnology, Harbin Veterinary Research Institute, Chinese Academy of Agricultural Sciences, Harbin, Heilongjiang 150001 China; 3 College of Veterinary Medicine, Northeast Agricultural University, Harbin Heilongjiang, 150030 China; 4 Chongqing Public Health Medical Center, Chongqing, 400038 China; 5 Comparative Immunology Research Center, College of Veterinary Medicine, China Agricultural University, Beijing, 100193 China; 6 Biomedical Analysis Center, Third Military Medical University, Chongqing, 400038 China; 7 Cancer Center, The General Hospital of Western Theater Command, Chengdu, Sichuan 610083 China; 8 Institute of Human Virology, Zhongshan School of Medicine, Sun Yat-sen University, Guangzhou, Guangdong 510080 China; 9 Institute for Hepatology, National Clinical Research Center for Infectious Disease, Shenzhen Third People’s Hospital, Shenzhen, Guangdong 518112 China; 10 NHC Key laboratory of Systems Biology of Pathogens, Institute of Pathogen Biology, Chinese Academy of Medical Sciences and Peking Union Medical College, 100176 Beijing, China

**Keywords:** Viral infection, Translational immunology

According to the World Health Organization (WHO) newly updated situation report on March 18th, 2020, the coronavirus disease 2019 (COVID-19) pandemic has confirmed 191,127 cases and claimed 7807 deaths worldwide.^1^ The etiological agent of COVID-19 has been identified as a novel coronavirus, the severe acute respiratory syndrome coronavirus 2 (SARS-CoV-2), belonging to *Sarbecovirus* subgenus (genus *Betacoronavirus*, family *Coronaviridae*) and showing 79.6 and 96.2% sequence identity in nucleotide to SARS-CoV and a bat coronavirus (BatCoV RaTG13), respectively.^2–4^ Like SARS-CoV infection, a substantial fraction of COVID-19 patients exhibits severe respiratory symptoms and has to be hospitalized in intensive care unit.^5–8^ Although the mortality rate of COVID-19 is significantly lower than that of SARS-CoV infection, SARS-CoV-2 shows much higher human-to-human transmission rate, rapidly leading to a global pandemic declared by WHO on March 11th, 2020.^9^

Currently, there are no approved prophylactic vaccines or therapeutic drugs that are specific to COVID-19. Blocking monoclonal antibodies (mAbs), due to their extraordinary antigen specificity, are one of the best candidates for neutralizing virus infection.^10,11^ Therefore, identifying and cloning blocking mAbs that can specifically target surface viral proteins to block the viral entry to host cells is a very attractive approach for preventing and treating COVID-19, in particular when effective vaccines and therapeutics are unavailable in the outbreak of the COVID-19 pandemic. We then sought to identify and clone blocking mAbs from the memory B cell repertoire of recently recovered COVID-19 patients to prevent the entry of COVID-19 virus to the host cells.

Similar to SARS-CoV, SARS-CoV-2 also utilizes highly glycosylated homotrimeric spike (S) protein for receptor binding and virus entry.^3,12–15^ The S protein of SARS-CoV-2 consists of two subunits, S1 and S2. To engage host cell receptor human angiotensin-converting enzyme 2 (hACE2), shared by both SARS-CoV and SARS-CoV-2, S protein undergoes dramatic conformational changes to expose the RBD and key residues for receptor binding. S protein is metastable, and binding of RBD to hACE2 receptor likely leads to the shedding of S1 protein from S2 protein, thus promoting S2-mediated virus-host membrane fusion and virus entry.^16–18^ Given the critical role of the RBD in initiating invasion of SARS-CoV-2 into host cells, it becomes a vulnerable target for neutralizing antibodies. Thus far, the human mAbs specifically target the SARS-CoV-2 RBD-hACE2 interaction have not been reported, and a monoclonal antibody targeting S1 made from immunized transgenic mice expressing human Ig variable heavy and light chains has been recently shown to neutralize both SARS-CoV-2 and SARS-CoV infection, but by an unknown mechanism that is independent of the blockade of RBD-hACE2 interaction.^19^

Prior to cloning SARS-CoV-2 RBD-specific human mAbs, we first examined whether patients recently recovered from COVID-19 had mounted anti-SARS-CoV-2 S1 protein IgG antibodies in sera. Among 26 recovered COVID-19 patients, we found that the majority of these recruited patients were able to produce high titers of SARS-CoV-2 S1-specific IgG antibodies and only three patients mounted relatively lower anti-S1 IgG responses, by enzyme-linked immunosorbent assay (ELISA) (Fig. 1a). Consistently, we also found that SARS-CoV-2 RBD-specific IgG antibodies were present in sera of all patients by ELISA (Fig. 1b). Next, we sought to investigate whether RBD-specific antibodies in patient serum can block the binding of SARS-CoV-2 RBD to hACE2. To this end, we set up an ELISA-based inhibition assay to examine the blocking function of these antibodies. We noted that there were only 3 out of 26 patients showed effective blockade of SARS-CoV-2 RBD binding to hACE2 (Fig. 1c). Taken together, these results suggested that while all recovered COVID-19 patients can generate anti-S1 and anti-RBD antibodies, there were only a small fraction of these antibodies can block the binding of RBD to hACE2 receptor. This observation may be explained by transient and dynamic perfusion conformational states of S protein that provide a very limited window for the immunogenic epitopes of RBD exposure to specific B cells.^20^

**Fig. 1:**
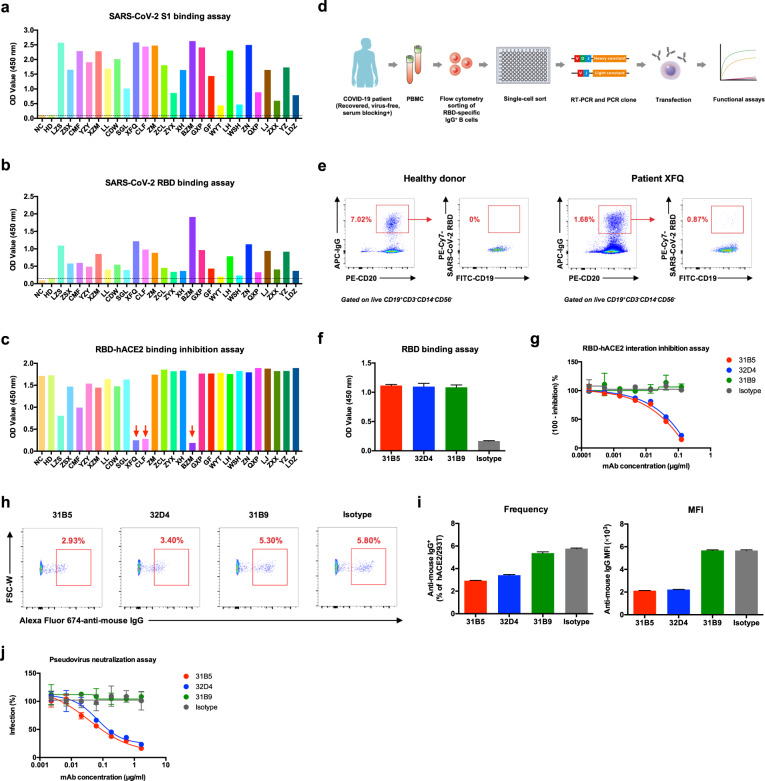
Human monoclonal antibodies block the SARS-CoV-2 RBD protein-hACE2 protein interaction **a** ELISA binding assay of COVID-19 patient sera to ELISA plate coating of SARS-CoV-2 S1 protein. **b** ELISA binding assay of COVID-19 patient sera to ELISA plate coating of SARS-CoV-2 RBD protein. **c** COVID-19 patient serum-mediated inhibition of the SARS-CoV-2 S1 protein binding to hACE2 protein by ELISA. **d** An overall strategy of anti-SARS-CoV-2 RBD mAbs. **e** Flow cytometry analysis of SARS-CoV-2 RBD-specific IgG^+^ B cells in PBMCs of healthy donor and patient XFQ. **f** Specificity of mAbs (311mab–31B5, −32D4 and −31B9 clones) to SARS-CoV-2 RBD protein by ELISA. **g** ELISA analysis of SARS-CoV-2 RBD-hACE2 interaction inhibited by 311mab–31B5, −32D4, and −31B9 mAbs. **h** Flow cytometry analysis of SARS-CoV-2 RBD-hACE2 interaction inhibited by 311mab–31B5, −32D4, and −31B9 mAbs. The numbers adjacent to the outlined areas indicate the percentages of anti-mouse IgG^+^ hACE2-plasmid transiently transfected 293T cells, which are summarized in **i** (left panel)**. i** (right panel) Mean fluorescence intensity (MFI) of Alexa Fluor 647 anti-mouse IgG in anti-mouse IgG^+^ hACE2-plasmid transiently transfected 293T cells. **j** Antibody-mediated blocking of luciferase-encoding SARS-Cov-2 typed pseudovirus into hACE2/293T cells. NC, negative control. HD, healthy donor. The data are representative of two independent experiments with three replicates per group (f, g, i, j; error bars in f, g, i, j indicate the SD)

Next, we set out to clone human mAbs using the blood samples from three COVID-19 recovered patients, of which their sera showed potent hACE2 receptor binding inhibition. To this end, we sorted each SARS-CoV-2 RBD-specific, IgG class-switched memory B cell into a single well of the 96-well microplates. Subsequently, we used reverse transcription polymerase chain reaction to amplify IgG variable heavy chain (VH) and light chain (VL) from each single memory B cell. After cloning both VH and VL, we inserted both sequences into expression plasmids that encoding constant regions of human IgG1 heavy chain and light chain (Fig. 1d).^21^ We found that SARS-CoV-2 RBD-specific, IgG-positive memory B cells only enriched in COVID-19 recovered patients, but not in healthy controls (Fig. 1e), suggesting the specificity of our sorting strategy. After antibody cloning, we acquired three pairs of IgG VHs and VLs inserted expression plasmids.

Finally, we expressed these paired plasmids encoding IgG VH and VL sequences and named these three mAbs as 311mab–31B5, 311mab–32D4 and 311mab–31B9, respectively. We first examined whether these human mAbs were able to bind to SARS-CoV-2 RBD protein by ELISA. The results showed that all three mAbs strongly and specifically bind to the RBD protein (Fig. 1f). Next, we tested whether these mAbs can block the interaction between SARS-CoV-2 RBD and hACE2. We found that both 311mab-31B5 and 311mab-32D4 could efficiently block SARS-CoV-2 RBD-hACE2 interaction (IC_50_ = 0.0332, and 0.0450 μg/ml, respectively), while 311mab–31B9 clone failed to inhibit such an interaction (Fig. 1g). The 31B5- and 32D4-mediated inhibition of RBD-hACE2 interaction was also evidenced by flow cytometry analysis (Fig. 1h, i). Furthermore, we determined the neutralization of these three mAbs using a SARS-CoV-2 S pseudotyped lentiviral particle.^22^ In line with ELISA- and flow cytometry-based blockade results, both 311mab-31B5 and 311mab-32D4 effectively neutralized pseudovirus entry to host cells ectopically expressing hACE2 (IC_50_ = 0.0338, and 0.0698 μg/ml, respectively). As expected, 311mab-31B9 clone failed to show any neutralization activities (Fig. 1j).

In conclusion, we have successfully cloned two human blocking mAbs using SARS-CoV-2 RBD-specific memory B cells isolated from recovered COVID-19 patients. These two mAbs can specifically bind to SARS-CoV-2 RBD, block the interaction between SARS-CoV-2 RBD and hACE2 receptor, and lead to efficient neutralization of SARS-CoV-2 S protein pseudotyped virus infection. Such human anti-SARS-CoV-2 RBD-hACE2 blocking mAbs are first reported, and hold great promise to be exploited as specific prophylactic and therapeutic agents against ongoing SARS-CoV-2 pandemic.

## Supplementary information

Materials and Methods
