# Supplementary material for: Human monoclonal antibodies block the binding of SARS-CoV-2 spike protein to angiotensin converting enzyme 2 receptor
Source: Cell Mol Immunol. 2020 Apr 20;17(6):647–9. doi: 10.1038/s41423-020-0426-7 (PMC7167496; doi:10.1038/s41423-020-0426-7)
Supplement: Supplementary file 1 — Materials and Methods [file 41423_2020_426_MOESM1_ESM.pdf]

Additional information

Materials and methods

Human samples. The 26 COVID-19 patients enrolled in the study were provided written informed consent. Prior to blood collection, the patients were clinically recovered and tested negative qPCR for SARS-CoV-2 virus RNA. Healthy control subjects were 2 adult participants in the study. The study received IRB approval at Chongqing Public Health Medical Center (2020-023-01-KY).

PBMC and serum collection. Blood samples were collected in cell preparation tubes with or without sodium citrate (BD Bioscience). PBMCs were isolated from blood in sodium citrate tubes using Ficoll (TBD Science), washed with PBS plus 2% FBS, suspended in cell freezing medium (90% FBS plus 10% DMSO), frozen in freezing chamber first at -80°C, and then transferred to liquid nitrogen. Sera were collected from blood without sodium citrate treatment and stored in aliquots at -80°C.

Single-cell sorting by flow cytometry. For B cell enrichment, PBMCs were firstly stained with FITC-conjugated anti-CD19 antibody (Biolegend) on ice for 30 min. Then, FITC-CD19 stained PBMCs were enriched using anti-FITC MicroBeads (Miltenyi Biotec) by following manufacturer's protocol. For surface staining, the enriched CD19^{+} B cells were stained with biotin-conjugated SARS-CoV-2 RBD protein (Sino Biological, 40592-V05H) at 4°C for 20 min, followed by PE-Cy7-conjugated streptavidin (eBioscience), PE-conjugated anti-CD20 antibody (Biolegend), APC-conjugated anti-human IgG (Fc) (Biolegend), APC-Cy7-conjugated anti-CD3 antibody (Biolegend), APC-Cy7-conjugated anti-CD14 antibody (Biolegend), APC-Cy7-conjugated anti-CD56 antibody (Biolegend) and APC-Cy7-conjugated LIVE/DEAD dye (Life Technologies). Cell staining was performed in PBS containing 5% mouse serum (wt/vol). For cell sorting, the stained SARS-CoV-2 RBD-specific IgG^{+} B cells were

single-cell sorted into 96-well plates loaded with 10 µl catch buffer and then stored at -80°C. Catch buffer: to 1 ml of RNAase-free water (Tiangen Biotech), add 50 µl 1.5 M Tris pH 8.8 (Beijing Dingguo Changsheng Biotech) and 40 µl Rnasin (NEB).

RT-PCR and PCR cloning. The heavy and light chain genes were PCR amplified as previously described.[21] Briefly, single-cell sorted plates were thawed on ice and added with 15 µl RT-PCR master mix following the one step RT-PCR kit protocol (Takara, RR057A) with primers for IgG VH and IgG VL. RT-PCR program: 50°C for 30 min, 94°C for 2 min, 45 cycles of 94°C for 30 sec, 57°C for 30 sec and 72°C for 1 min. Then, RT-PCR products were nested PCR-amplified with nested PCR master mix following the HS DNA polymerase kit protocol (Takara, TAK R010) with primers for IgG VH or IgG VL. Nested PCR program: 98°C for 4 min, 45 cycles of 98°C for 1 min, 57°C for 1 min and 72°C for 1 min. Next, heavy and light chain PCR products were purified and nuclease digested with Age1-HF/Sal1-HF (NEB) and Age1-HF/BsiW1-HF (NEB), respectively. The digested heavy and light chain genes were further cloned into human IgG1 heavy chain and light chain expression vectors, respectively.

Transfection. Human embryonic kidney (HEK) 293T cells of 80-90% confluent in the 15 cm tissue culture plate were transfected with master mixture containing 9 µg heavy chain plasmid, 9 µg light chain plasmid and 60 µl *Trans*IT-293 Transfection reagent (Mirus). The culture media was changed to basal media 24 hours-post transfection. Then, the culture media was collected from the plate 2 days later.

ELISA. 50 ng of SARS-CoV-2 S1 protein (Sino Biological, 40591-V08H) or SARS-Cov2 RBD protein (Sino Biological, 40592-V08B) in 100 µl PBS per well was coated on ELISA plates overnight at 4°C. Then, the ELISA plates were blocked for 1 hour with blocking buffer (5% FBS plus 0.05% Tween 20). Next, mAbs or ten-fold diluted patient sera were added to each well in 100 µl blocking buffer for 1 hour. After

washing with PBST, the bound antibodies were incubated with anti-human IgG HRP detection antibody (Bioss Biotech) for 30 min, followed by washed with PBST, then PBS and addition of TMB (Beyotime). The ELISA plates were allowed to react for 5 min and then stopped by 1 M HCl stop buffer. The optical density (OD) value was determined at 450 nm.

ELISA-based receptor-binding inhibition assay. 200 ng of hACE2 protein (Sino Biological, 10108-H08H) in 100 μl PBS per well was coated on ELISA plates overnight at 4°C. Then, the ELISA plates were blocked for 1 hour with blocking buffer (5% FBS plus 0.05% Tween 20); meanwhile, three-fold serial dilutions of mAbs or ten-fold diluted patient sera were incubated with optimal dose (based on EC_{50}) of SARS-Cov2 RBD protein (Sino Biological, 40592-V05H) for 1 hour. Then, the incubated mixtures were added to ELISA plates and allowed to develop for 30 min, followed by PBST washing and anti-mouse Fc HRP antibody (Thermo Fisher Scientific). Next, the ELISA plates were washed with PBST, then PBS and added with TMB (Beyotime). After 5 min, the ELISA plates were stopped and determined at 450 nm. The half maximal inhibitory concentration (IC_{50}) was determined by using 4-parameter logistic regression.

Flow cytometry-based receptor-binding inhibition assay. 311mab mAbs or isotype were incubated with optimal dose (based on EC_{50}) of SARS-Cov2 RBD protein (Sino Biological, 40592-V05H) for 1 hour at RT. Then, the mixtures were incubated with 10,000 hACE2-plasmid transiently transfected 293T cells for 40 min on ice, followed by stained with Alexa Fluor 647-conjugated goat anti-mouse IgG (Biolegend) and APC-Cy7-conjugated LIVE/DEAD dye (Life Technologies).

Pseudovirus neutralization assay. Spike protein of SARS-Cov-2 typed pseudovirus was produced as previously described.[22] Concisely, HEK-293T cells were transfected

with psPAX2, pLenti-GFP and 2019-nCov S plasmids by using *Trans*IT-293 Transfection reagent (Mirus). The culture media was changed to fresh media 12 hours-post transfection. And at 64 hours after transfection, supernatants were harvested. For pseudovirus neutralization assay, three-fold serially diluted mAbs were mixed with SARS-Cov-2 typed pseudovirus for 1 hour. Then, the mixture was incubated with hACE2-expressing HEK-293T (hACE2/293T) cells overnight, followed by change of fresh media. At 40 hours-post incubation, the luciferase activity of infected hACE2/293T cells were detected by Dual-Luciferase Reporter Assay System (Promega). The percent of infection was calculated as ratio of luciferase value with mAbs to that without mAbs. The half maximal inhibitory concentration (IC_{50}) was determined by using 4-parameter logistic regression.
